# Malignancies in Patients with Celiac Disease: Diagnostic Challenges and Molecular Advances

**DOI:** 10.3390/genes14020376

**Published:** 2023-01-31

**Authors:** Mariia Ivanova, Luca Bottiglieri, Elham Sajjadi, Konstantinos Venetis, Nicola Fusco

**Affiliations:** 1 Division of Pathology, IEO, European Institute of Oncology IRCCS, 20141 Milan, Italy; 2 Department of Oncology and Hemato-Oncology, University of Milan, 20122 Milan, Italy

**Keywords:** celiac disease, cancer, gastrointestinal disease, biomarkers, diagnosis, molecular profiling, omics, gut microbiota

## Abstract

Celiac disease (CD) is a multiorgan autoimmune disorder of the chronic intestinal disease group characterized by duodenal inflammation in genetically predisposed individuals, precipitated by gluten ingestion. The pathogenesis of celiac disease is now widely studied, overcoming the limits of the purely autoimmune concept and explaining its hereditability. The genomic profiling of this condition has led to the discovery of numerous genes involved in interleukin signaling and immune-related pathways. The spectrum of disease manifestations is not limited to the gastrointestinal tract, and a significant number of studies have considered the possible association between CD and neoplasms. Patients with CD are found to be at increased risk of developing malignancies, with a particular predisposition of certain types of intestinal cancer, lymphomas, and oropharyngeal cancers. This can be partially explained by common cancer hallmarks present in these patients. The study of gut microbiota, microRNAs, and DNA methylation is evolving to find the any possible missing links between CD and cancer incidence in these patients. However, the literature is extremely mixed and, therefore, our understanding of the biological interplay between CD and cancer remains limited, with significant implications in terms of clinical management and screening protocols. In this review article, we seek to provide a comprehensive overview of the genomics, epigenomics, and transcriptomics data on CD and its relation to the most frequent types of neoplasms that may occur in these patients.

## 1. Introduction

Celiac disease (CD) is an autoimmune multiorgan disorder triggered by dietary gluten, characterized by chronic enteropathy in genetically predisposed individuals [1]. Its treatment relies on a gluten-free diet [2,3]. The pooled global prevalence of this condition has increased over the past 50 years, ranging from 0.7% (histopathological diagnosis) to 1.4% (seroprevalence) to date [4].

The diagnosis of CD relies on the combination of clinical, serological, and histopathological findings [1]. Although the recommended first-line diagnostic serological test with IgA tissue transglutaminases (TTA) shows a high sensitivity and specificity, with the evolution of endoscopic and biopsy techniques, histopathological evaluation has become a cornerstone [5,6,7]. The main histopathologic features of CD include the elevated number of intraepithelial T lymphocytes, villous atrophy, crypt hyperplasia, and decreased enterocyte height [1,2,7,8]. The histological interpretation of small-bowel biopsy should be essentially conducted in strong collaboration with a gastroenterologist for the establishment of clinical–pathological correlations [1,6]. Biopsy-confirmed CD is 1.5 times more common in females than in males, and approximately twice more common in children than in adults, with higher prevalence in Caucasian populations [4]. However, the lack of unbiased population-based studies in many countries prevents the proper establishment of the exact global burden of CD. The typical clinical manifestations of CD are related to malabsorption and include abdominal pain, steatorrhea, and diarrhea [9]. However, the spectrum of CD-related symptoms is extremely wide, comprising heterogeneous conditions, such as gastroesophageal, cardiovascular, neurologic, and endocrine disorders [9,10,11]. Lately, a significant number of studies have considered the possible association between CD and neoplasms [8]. Indeed, a higher mortality rate in patients with CD is associated with several types of malignancies [8,12]. However, the role of CD in increasing (or decreasing) the risk of cancer remains a matter of great controversy.

In this review article, we seek to provide the current evidence in the literature about the possible development of cancers in patients with a diagnosis of CD. A comprehensive review of the literature is carried out and each tumor type is analyzed separately. We also aim to review and analyze current screening, treatment, and prevention strategies, adding our opinion to possible future prospectives.

## 2. Immunogenetics and Comorbidities

Understanding CD pathogenesis has extended beyond the traditional concept of a purely autoimmune disorder, being at present considered as a dynamic process of small intestine mucosal remodeling due to a spectrum of immunologic processes [7,13]. These are based on various components, such as neutrophils, eosinophils, mast cells, and complement, which contribute to disease pathogenesis and evolution [7,14,15]. The main genetic predisposing factors for CD are in the major histocompatibility complex (MHC) region, which is located on chromosome 6p21, comprising several immune-related genes [16]. In this respect, it has been proposed that CD may predispose to certain cancer types due to persistent immune activation [8,17]. Hence, CD and cancer share some hallmarks [18], such as inflammation, genome instability and mutations [19], phenotypic plasticity [13], epigenetic reprogramming [20], and polymorphic microbiomes [21]. In particular, chronic inflammation is one of the key aspects of CD and one of the acknowledged cancer causes [17,22,23]. Thus, a well-known association of CD with *HLA-DQ2* genes was first identified in 1989 [24], and *HLA-DQA1* and *HLA-DQB1* genetic variants are known to account for up to 48% of disease etiology [25]. The prevalence of these HLA haplotypes in the general population is around 30–40%, suggesting that they are not sufficient to induce CD on their own [20]. No other genetic factors were identified for a long time until the comprehensive genomic profiling era was established, with one of the biggest clinical studies featuring the recruitment of 1048 biopsy-proven coeliac disease patients [26,27,28]. This allowed researchers to uncover a numerous amount of non-*HLA* genetic markers and differentially expressed genes, improving the understanding of CD pathophysiology and resulting in over 550,000 single-nucleotide polymorphisms (SNPs) genotyped to date [25,26,28]. However, most of the identified variants are located in non-coding regions of the genome, making the interpretation of their functional role challenging [26]. To date, it has been possible to explain only up to 55% of CD heritability, suggesting numerous genetic variants with minor allele frequencies below 5–10% that may not have been taken to account [26].

Overall, CD patients show an increased prevalence of autoimmune diseases and present an overlap of genes with Crohn’s disease, ulcerative colitis, type 1 diabetes, rheumatoid arthritis, and systemic lupus erythematosus (SLE), which potentially may contribute to tumorigenesis [6,26,27,29,30,31,32,33,34]. These results have been collected in different numbers of patients, starting from case studies of a single case to large retrospective and prospective longitudinal studies and dataset analysis [6,26,27,29,30,31,32,33,34]. One of the largest analyses of the main Genome-Wide Association Studies (GWAS) carried out in 2021 pinpointed the importance of the discovery of thousands of genetic polymorphisms and genome variations that underlie the risk of different diseases, including CD [26,35]. Publicly available datasets, analyzed by Inamo, included GWAS for CD (featuring 12,041 cases and 12,228 controls) of the European population as the exposure and GWAS for SLE (1311 cases and 1783 controls) of the European population as the outcome [27]. For example, the discovery of *KIAA1109-TENR-IL2-IL21* block on chromosome 4 contains the well-known immune disease *IL2-IL21* locus and was the first non-*HLA* risk locus associated with CD [26,36]. At present, many known genes that have been identified in families with CD belong to interleukin family signaling and immune-related pathways (*CD3E, FBXL7, PSMA8,* and *PPP2R1B*), while two genes (*PSMA8* and *PPP2R1B*) play a role in the innate immune response pathway and *IL1R1*, *PPP2R1B*, and *PSMA8* are involved in the interleukin signaling (IL-1, IL-10, and IL-17) pathway [25]. Remarkably, an increase in *PSMA8* expression has been reported in different tumors, such as large B-cell lymphoma, thymomas, and testicular germ cell tumors [37].

A distinct subtype of CD, refractory CD (RCD), has been described, where patients’ symptoms fail to improve regardless of strict gluten-free diet implementation, thus leading to the progress of villous atrophy [9,38]. It is mostly diagnosed in patients over 50 years of age and the range is 0.04–1.5% [38]. Common histological findings include chronic inflammation and crypt hypoplasia with villous atrophy [9]. This CD type is divided into two subtypes, type 1 (RCD-1) and type 2 (RCD-2), where the latter features abnormal intraepithelial lymphocytes count and mutations in genes shared by some cancer types, such as Janus kinase 1 (*JAK1)* or Signal transducer and activator of transcription 3 (*STAT3)*, which play role in the nuclear factor kappa light chain enhancer of activated B cells (NF-κB) pathway, triggering CD-associated lymphomagenesis in CD patients [1,38,39,40,41].

The newest study by Atlasy et al. conducted a single-cell transcriptomic analysis of the immune cell compartment in CD and revealed five distinct immune-cell compartments in the lamina propria of the human small intestine by single-cell RNA-sequencing analysis and an increased number of proinflammatory macrophages in CD associated with interferon-gamma signaling [42,43]. Overall, recent advances in genetic and epigenetic fields may contribute to a better understanding of the disease pathophysiology and a better diagnosis of CD.

### 2.1. Gut Microbiota

The gut microbiota is constituted by the collective of microorganisms (bacteria, archaea, eukaryotes, and viruses) populating the intestinal tract, providing aid in digestion, vitamin production, and balancing the immune and metabolic microenvironment [20,26]. Although bacterial dysbiosis has been widely recognized as an important feature of irritable bowel syndrome (IBS) and colorectal cancer [44,45,46,47,48], some studies conducted analyses of fungal and viral microbiome and claim it should not to be overlooked [44,49,50,51,52].

Longitudinal multi-omics analyses of IBS diseases included CD patients and aimed to study microbiome, metabolome, and epigenome of the subjects. The data identified that all patients with IBS have an increased bacterial metabolite tryptamine, which stimulates colonic mucosal secretion and immune activation via inflammatory-related pathways [53,54].

The role of gut microbiota has been suggested to play a role in CD development [20,26]. In particular, the *HLA-DQA1* and *HLA-DQB1* alleles are believed to affect the gut microbiota composition and were found to be predictive of a favorable response to a gluten-free diet in patients with IBS [55,56]. Current studies revealed a decrease in Bifidobacteria and increase in Bacteroides numbers in patients with CD, while infants with a genetic predisposition have an abundance of Proteobacteria and Firmicutes [20,26,57,58]. One of the largest ongoing prospective longitudinal multi-center CDGEMM Study (NCT02061306) is recruiting 500 infants with a first-degree family member diagnosed with CD to address genomic, environmental, microbiome, and metabolomic factors that could affect the development of CD [21]. The primary outcome of this study is represented by the measurement of the change in microbiota composition of CD in at-risk infants using culture-independent high-throughput sequence analysis of the 16S rRNA genes [21,59]. The preliminary results of this study suggest that individual metabolomic phenotypes, as a result of gene–diet–gut microbiome interactions, can help to define specific enterotypes associated to gluten tolerance loss in infants genetically at risk of CD [21].

The main limitations of most available studies include missing genetic associations and limited cohort numbers [26].

### 2.2. DNA Methylation

DNA methylation studies have been shown to be relevant in different diseases and cancer types, and CD-related DNA methylation was first described in 2010 [26,60,61,62,63]. Ultimately, the allele-specific DNA methylation (ASM) combined with comprehensive genomic profile data has shown the presence of CD-specific SNPs, which can nominate specific transcriptional pathways in CD and represent possible targets in disease management [63]. Overall, ASM contributes significantly to the discovery of the diseases’ epigenomics, identifying previously unknown SNPs to fulfill the understanding of the diseases [26,63].

The first genome-wide methylation study has been published in 2019, defining 43 and 310 differently methylated positions in epithelial and immune fractions, respectively [64]. According to this study, the loss of CpG island (CGI) borders, which is frequently linked to altered gene expression, and the increased methylation variability might provide a substrate for defining the epithelial methylome of these patients. Few CD-associated SNPs or variations that contribute to methylation quantitative trait loci (mQTLs) align with differentially methylated locations (DMPs). These findings validate the role of DNA methylation changes in the HLA region and support the contention that they are a genotype-independent event.

A comprehensive gene expression in CD has been studied at the level of the entire intestinal mucosa (epithelium and lamina propria). As a result, the reverse modulation of gene expression and methylation in the same cellular compartment was observed for the *IL21* and *SH2B3*, which led the authors to suggest that a “gene-expression phenotype” of CD and the abnormal response to dietary antigens in CD might be related to the regulation of molecular pathways, and not gene alterations [65]. The same authors previously have defined a small set of candidate genes in peripheral blood mononuclear cells that was able to predict CD at least 9 months before the appearance of any clinical and serological signs of the disease, which could develop into a potential non-invasive epigenetic instrument of screening instead of intestinal biopsy [66]. Altered DNA methylation profiles appear to be present in the saliva of CD individuals, which may be helpful in the development of non-invasive diagnostic methods [67].

### 2.3. MicroRNA

MicroRNAs (miRNAs) are short RNA sequences, regulating transcription factor, gene expression at the post-transcript level, and the translation of protein-coding genes [20,26]. They are strongly implicated in the pathogenesis of many diseases, including inflammatory bowel diseases and CD [68,69]. It has been confirmed the regulatory role of miRNAs on cell proliferation in CD and several studies have identified different subsets of miRNAs allowing researchers to stratify CD patients by the severity of intestinal damage [20,70,71]. A significant miR-31-5p downregulation has been noted in CD, and miR-192-5p and miR-192-3p were downregulated in CD patients with severe histological lesions and anemia, respectively [72]. miR-192-5p has been found to target two molecules, NOD2 and CXCL2, involved in innate immunity, which were upregulated in severe cases of CD. This miRNA targets FOXP3, which is essential for regulatory T-cell development. A significant inverse correlation was observed between the miRNA and the target mRNA, and interestingly, discovered miR-192 are similar to those observed in inflammatory bowel disease [20,69,71].

Tan et al. applied next-generation sequencing, correlating miRNA and mRNA expression patterns to generate a CD-specific transcript interaction network. Various pathways have been shown to be deregulated, such as barrier homeostasis, lipid metabolism, and immunity (interferon signaling), a key factor in the pathophysiology of CD, suggesting miRNAs play a key role in the intestinal damage [69].

Another possible area of application may be a study of circulating miRNAs as a non-invasive diagnostic alternative. The upregulation of miR-21 and downregulation of miR-31 expression in active CD patients compared to the treated ones has been demonstrated in pediatric patients and the positive correlation between miR-21 expression and IgA auto-antibodies against tissue transglutaminase has been observed, which is a major auto-antigen in CD [73].

Future investigations are needed to explore the miRNA roles in CD pathogenesis and their potential role as biomarkers, with a larger validation cohort, and probably in comparison with other inflammatory bowel diseases [20,26,69].

## 3. Risk of Malignancies in Patients with Celiac Disease

One of the latest nationwide cohort studies in Sweden reports that patients with CD are, overall, at an increased risk of developing malignancies, especially those diagnosed with CD after the age of 40, with a particular predisposition for lymphoma, oropharyngeal, and intestinal cancer [2,8,12,17,74,75,76]. Lebwohl et al. studied 47,241 CD patients, demonstrating an increased risk of cancer incidence after a median follow-up of 11.5 years compared to the control group (Hazard ratio (HR), 1.11; 95%; confidence interval (CI), 1.07–1.15), being significantly elevated in the first year after CD diagnosis (HR, 2.47; 95% CI, 2.22–2.74), higher in patients diagnosed in the age range of 40–59 years old (HR, 1.07; 95% CI, 1.01–1.14), and the highest in patients diagnosed with CD after the age of 60 years (HR, 1.22; 95% CI, 1.16–1.29) [12]. Overall, men with CD had a higher cancer risk than women [12].

**Figure 1:**
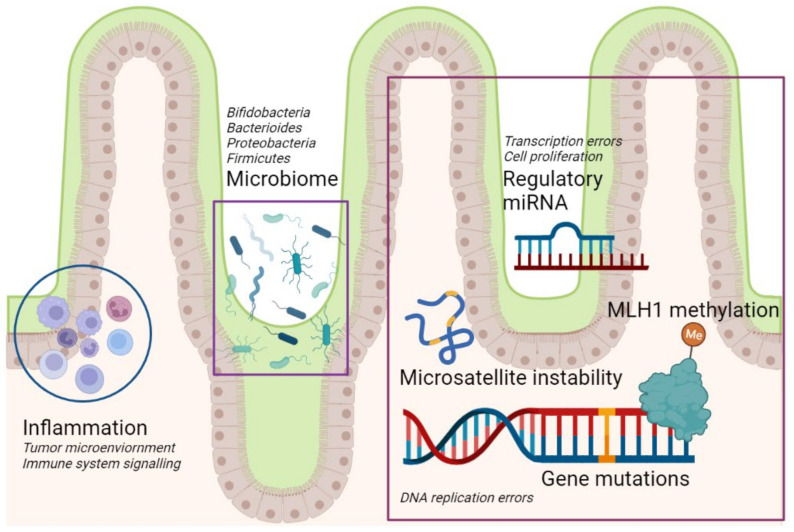
Schematic representation of the cancer hallmarks commonly shared with alterations found in celiac disease, featuring: possible microbiome alterations; the role of inflammation and tumor microenvironment; role of regulatory miRNAs, leading to transcription and cell proliferation errors if impaired; and microsatellite instability with MLH1 promoter hypermethylation, leading to DNA replication errors. MLH1, DNA mismatch repair protein MutL protein homolog 1. Created with BioRender.

The schematic representation of the cancer hallmarks commonly shared with alterations found in celiac disease that can potentially contribute to cancer development in CD patients is reproduced in Figure 1.

### 3.1. Lymphoproliferative Disorders 

Exploring the types of cancer present in the cohort, the strongest association between CD has been for hematologic neoplasms [77]. Refractory celiac disease type 2 (RCD-2), also referred to as "cryptic" enteropathy-associated T-cell lymphoma or "intraepithelial T-cell lymphoma", has been found to be a rare clonal lymphoproliferative disorder arising from innate intraepithelial lymphocytes. It is known to have a poor prognosis and frequently evolves to enteropathy-associated T-cell lymphomas (EATL) if CD is untreated [77].

The risk of EATL was strongly associated with CD diagnosis (RR = 35.8 (95% CI, 27.1–47.4)), according to the Dutch nationwide population-based pathology database (PALGA) [78,79]. Proposed mechanisms of CD association with lymphoma include the effects of chronic inflammation and antigen-driven T-cell proliferation [12,17,32,80]. Interestingly, the risk of lymphoproliferative malignancies was increased in CD patients and in those with inflammation (HR, 2.82; 95% CI, 2.36–3.37 and 1.81; 95% CI, 1.42–2.31, respectively); however, in subjects with only positive serology, a risk of lymphoma development was similar to that of the general population (HR, 0.97; 95% CI, 0.44–2.14) [75]. Multiple studies confirm the previously obtained data where CD patients with persistent villous atrophy are at an increased risk of lymphoproliferative malignancies, especially EATL, compared to the general population (SIR, 3.78; 95%, CI, 2.71 to 5.12), although the mechanism is not very well understood [12,17,32,80,81,82,83]. The genetic alterations described in RCD II include epigenetic regulators, DNA damage repair, immune evasion genes, mutations of the tumor suppressors tumor necrosis factors alpha-induced protein 3 (TNFAIP3), and receptor superfamily member 14 (TNFRSF14) alterations [39,84]. Of note, deep deletions and truncating mutations of both these tumor suppressors are recurrent in non-Hodgkin lymphomas and leukemias [85]. The adherence to a gluten-free diet has shown an effective inhibition of EATL [32,86]. Other non-Hodgkin lymphomas (NHL) have also been demonstrated to be of a higher incidence in celiac patients, where T-cell lymphomas are enteropathy-associated and B-cell lymphomas are more likely to develop in patients with CD and dermatitis herpetiformis [76,87,88]. Gao et al. conducted a study of 37,869 NHL patients and stated a risk of >5 times higher in CD patients compared to CD-unaffected controls (OR = 5.35; 95% CI, 3.56–8.06) [75,89]. Somatic mutations of *STAT3* typical for RCD-2 are noted to occur in 10.4% of mature B-cell neoplasms, and common genes shared with Crohn’s disease are expressed in diffuse large B-cell lymphoma (*PTPN2, IL18RAP, TAGAP,* and *PUS10*) [90,91]. Another gene found to be mutated in RCD-2 and to trigger CD-associated lymphomagenesis was *JAK1* [1,38,39]. It has been recently demonstrated that 80% of RCD-2 and 90% of EATL display somatic gain-of-functions mutations in the *JAK1-STAT3* pathway, including a remarkable p.G1097 hotspot mutation in the JAK1 kinase domain in approximately 50% of cases, assuming the *JAK1-STAT3* pathway to be the main driver of CD-associated lymphomagenesis [39,92].

It has been previously reported that *JAK1* mediates autocrine IL-6 and IL-10 cytokine signaling in activated B-cell-like diffuse large B-cell lymphoma by a certain epigenetic regulatory mechanism involving phosphorylation of histone H3 on tyrosine 41 [93]. This observation suggests a new therapeutic strategy as JAK1 inhibitors synergize with inhibitors of active B-cell receptor signaling [93]. Of note, JAK1 inhibitors, targeting the JAK-STAT pathway, have been also regarded as a possible treatment for inflammatory bowel diseases [94,95]. Other putative drivers mutations in interferon regulatory factor 4 (IRF4) have been described in CD patients [96]. This transcription factor, which is involved in the differentiation of T and B lymphocytes, is altered by mutation and chromosomal rearrangement in various hematologic malignancies [85].

### 3.2. Head and Neck Carcinomas

Askling et al. have identified a higher risk of oropharyngeal cancer development in celiac patients by 2.3-fold [17,74]. The risk of oropharyngeal cancer has been linked to the higher incidence of gastroesophageal reflux caused by delayed stomach emptying and malabsorption [17,97,98].

Numerous CD-related genes have been found to be altered in head and neck cancers, such as *LPP* (lipoma-preferred partner), *SCHIP1* (schwannomin-interacting protein 1), and *IL12A* (interleukin-12 subunit alpha) [90,91]. The latter gene encodes a subunit of cytokine IL12 acting on T and NK cells, activating them, and this gene is upregulated in an experimental model of anticancer response, suggesting a possible antitumor mechanism [15]. Later studies have demonstrated the potential for intratumorally delivered IL12 mRNA to promote TH1 tumor microenvironment transformation and robust antitumor immunity [99,100].

Typical *STAT3* and *JAK1* gene alterations in RCD-2 are known to be activated in head and neck squamous cell carcinoma, representing an important therapeutic target that may be of a particular interest in CD patients with the abovementioned mutations [101,102].

### 3.3. Gastrointestinal Cancers

Exploring the types of cancer present in the Swedish cohort, a strong association between CD and gastrointestinal cancers (HR, 1.34; 95% CI, 1.24–1.45) has been observed. Among the gastrointestinal cancer subtypes, elevated risks have been observed for hepatobiliary cancer (HR, 1.80; 95% CI, 1.44–2.25) and pancreatic cancer (HR, 2.30; 95% CI, 1.87–2.82) but not for gastric cancer (HR, 1.21; 95% CI, 0.91–1.61) or colorectal cancer (HR, 1.06; 95% CI, 0.96–1.18) [12,103,104,105].

A meta-analysis of 17 studies from the biggest databases (Pubmed, Embase) by Han et al. has demonstrated that CD was associated with a 60% increase in GI cancer risk (pooled OR = 1.60, 95% CI 1.39–1.84), suggesting that CD patients had a higher risk of developing esophageal cancer with a pooled OR for esophageal cancer of 3.72 (95% CI, 1.90–7.28) [105]. Same authors found CD patients to be at a higher risk of small intestinal carcinoma (pooled OR = 14.41; 95% CI, 5.53–37.60), but without significant associations between CD and risk of gastric cancer (OR = 1.53; 95% CI, 0.96–2.44), colon cancer (pooled OR = 1.15; 95% CI, 0.86–1.56), or rectal cancer (OR = 0.90; 95% CI, 0.71–1.14) [105].

#### 3.3.1. Gastroesophageal Cancer

A 4.2-fold risk of developing esophageal cancers in CD patients has been previously reported [12,17,74,103,104,105]. A meta-analysis conducted by Han et al. indicates that esophageal cancer risk is higher in the peridiagnostic period (pooled OR = 4.02; 95% CI, 1.54–10.52) rather than postdiagnostically (pooled OR = 2.17; 95% CI, 1.34–3.51) [105], which is probably related to a higher frequency of endoscopic procedures in CD patients.

A case–control study from the Dutch nationwide population-based pathology database (PALGA) found that an increased CD-associated risk of esophageal squamous cell carcinoma was restricted to female patients and age over 50 at the time of diagnosis (RR = 5.9 (95% CI, 3.3–10.3) [78].

The risk of esophageal, and subsequently oropharyngeal cancer, was associated with a higher gastroesophageal reflux risk due to malabsorption and delayed stomach emptying, possibly complicated by reflux, which represents a risk of chronic inflammation and (pre)malignant epithelial changes [17,74,106]. Shared biological pathways have been observed with head and neck cancer [17,90,91,97].

The role of engulfment and cell motility protein 1 (ELMO1) in gastrointestinal cancer promotion has been widely discussed. ELMO1 is one of the key proteins for innate immunity, responsive of pathogenic bacteria and apoptotic cell clearance, regulating inflammatory responses by phagocytosis, reshaping, and cell migration promotion [107,108]. While its role in infectious process is well established, the role of ELMO1 in cancer still needs to be explored, as it may possibly trigger malignant cells’ invasion and metastasis [107,108].

The deregulation of ELMO1 has been found in inflammatory bowel disease, positively correlating with inflammatory cytokines expression, and has been proposed as a potential early biomarker [109].

The discovery of the deleterious effect of ELMO1 alterations in CD may provide a link in association to many gastrointestinal tumors, mainly esophageal and gastric cancer, which feature alterations of ELMO1 in up to 8%, and this gene has been proposed as a diagnostic or prognostic biomarker [90,91,107,110]. Moreover, ELMO1 alterations have been shown to play a role in HPV-related oropharyngeal squamous cell carcinoma, in metastatic spread of squamous cell carcinoma by means of TGFβ signaling, and epithelial-to-mesenchymal transition in gastric cancer [107,111].

Other altered genes, found in CD, have also been implicated in esophagogastric cancers pathways. These include *ATXN2* (Ataxin-2), which mediates the translation of TNFR1, promoting esophageal squamous cell carcinoma, or *ITGA4*, a cytoskeleton protein involved in gastric cancer cells migration [112,113,114]. These genes have been found altered in 3–4% of esophageal cancers [90,91]. *PSMA8* (Proteasome 20S subunit alpha 8), linked to innate immune response pathway and CD pathogenesis, was found altered in around 5% of esophageal cancers [90,91,112,113,114].

It is widely known that the reflux may also cause premalignant epithelial transformation (Barett’s esophagus), which is an additional risk factor, but all these risks have been shown to be successfully attenuated if a gluten-free diet was rigorously followed, [17,74,115] and reflux esophagitis itself, unless ulcered, is not an indication for esophageal biopsy [116].

#### 3.3.2. Small Bowel Cancer

Limited data are available on small bowel carcinoma (SBC), although a strong association of CD with this cancer type has been observed [17,117,118].

A retrospective study in Sweden has identified an increased risk of small bowel cancer in CD patients (HR, 3.05; 95% CI, 1.86–4.99) [92,119], which confirms the prior meta-analysis study (pooled OR = 14.41; 95% CI, 5.53–37.60), and, similarly to esophageal cancer, this risk is higher in the peridiagnosis period (pooled OR = 17.08; 95% CI, 3.59–81.20) compared to the postdiagnosis period (pooled OR = 4.64; 95% CI, 1.06–20.26) [105].

SBC is usually diagnosed at an advanced stage because of late-presenting symptoms [79]. In CD, it is characterized by a younger age of onset, a higher prevalence in the female gender, most frequent occurrence in jejunum, a higher prevalence of medullary type, and better overall survival compared to sporadic, Crohn- and hereditary syndrome-related SBC, which also represent a high level of microsatellite instability (MSI) [78,120]. It has been noted that SBC is most often synchronously diagnosed with CD, suggesting a probability of these patients having a silent CD before developing the symptoms of malignancy [105].

Chronic inflammation in CD has been shown to potentially contribute to the risk accumulation by enterocyte destruction potentiating premalignant changes [7,17,82,121], and some authors have found an association with a small bowel adenoma as a precursor lesion [117]. Epigenomic studies have demonstrated an *APC* promoter hypermethylation found in 73% of CD-associated small intestine malignancies, and this gene is frequently altered in small bowel and colorectal cancer types [60,90]. Vanoli et al. compared the histological and molecular features of small bowel carcinomas arising from patients with CD and Crohn’s disease (CrD), as both are potentially cancer-predisposing conditions [122]. CD patients have been found to harbor microsatellite instability with MLH1 promoter hypermethylation more often than CrD ones and have a higher number of tumor-infiltrating lymphocytes (TILs), which suggested a better outcome [120,122,123].

Another large Swedish cohort study of 48,119 patients with CD conducted by Emilsson et al. demonstrated a low risk of small bowel adenocarcinoma overall (HR 3.05; 95% CI, 1.86–4.99], but the risk of its incidence, as well as the risk of small bowel adenoma, was higher in CD patients compared to the healthy individuals [105,119]. Overall, delayed diagnosis, untreated CD, and persistent villous atrophy are the most frequently mentioned risk factors of malignant complications [79,119].

#### 3.3.3. Colorectal Cancer

A low risk of colorectal cancer in CD patients has been confirmed by an Italian cohort study of 1757 celiac patients, where only 6 patients developed colon carcinoma during the mean follow-up period of 18.1 years [103]. The standardized incidence ratio was 0.29 (95% CI=0.07–0.45), dropping to 0.07 (95% CI = 0.009–0.27) in CD patients with a strict adherence to a gluten-free diet [103].

However, Lasa et al. demonstrated a higher incidence of colorectal adenoma among celiac patients compared to the control group (47.37% versus 27.97%, *p* = 0.01), known to be a precursor lesion of colorectal cancer, with a particular increase in the prevalence of left-sided lesions [104,124]. Another study evaluated similarities in gut microbiota alterations, which may share common activation pathways in CD and colorectal cancer [125].

Although colorectal cancer is the third most prevalent malignancy in the world population [126], its lower risk in celiac patients may be explained by an overall healthier diet due to the certain product groups’ limitation and decreased capability of fat absorption, which attenuates most of the main inflammatory risk-factors involved in gastrointestinal tumor process [17,23]. The risk may be attenuated also by the lower body mass index (BMI) of celiac patients compared to healthy individuals, which is a crucial factor in colorectal cancer development [17,88]. Interestingly, a functional novel long non-coding RNA IQCJ-SCHIP1-AS1 has been shown to carry an indicative tumor-suppressor role and appears to be a potential prognostic factor in colorectal carcinoma, which may be significant in view of the known *SCHIP1* alterations in CD [127,128].

### 3.4. Hepatobiliary and Pancreatic Cancer

Among the gastrointestinal cancer subtypes, elevated risks were observed for hepatobiliary cancer (HR, 1.80; 95% CI, 1.44–2.25) and pancreatic cancer (HR, 2.30; 95% CI, 1.87–2.82) [12,103,104,105].

The higher proneness to hepatobiliary carcinomas in CD patients may be partially explained by liver enzymes’ disbalance and gut microbiota alterations with decreased Bifidobacterium quantities, resulting in an increased liver cancer risk [17]. In patients who do not strictly follow a gluten-free diet, an inflammation provoked by gluten ingestion may lead to excessive liver fibrosis and even cirrhosis, which is a known malignancy precursor [17,129]. Another possible explanation may be the average higher rice and corn consumption by these patients as gluten-free carbohydrate sources. It is known that rice, corn, and soybeans may contain aflatoxin, a mycotoxin produced by Aspergillus flavus and related fungus that contaminates foods due to impropriate storage. This toxin has been shown a major risk factor for hepatocellular carcinoma development [130,131].

As alterations of ELMO1, found in CD patients, are shown to have the effect on epithelial-to-mesenchymal transition; unsurprisingly, its levels are found to be elevated in hepatocellular carcinomas compared to adjacent non-tumor tissues [107,132]. This process was found to be mediated through PI3K/Akt pathway, which was confirmed by Gene Enrichment and Pathway (KEGG) analysis [132]. It has also been found that the TRIM27–USP7 complex promotes tumor progression via *STAT3* activation in human hepatocellular carcinoma, which could represent a possible therapeutic target in CD patients as well [133].

The development of pancreatic cancer has been shown to be linked to *PSMA8* alterations in up to 5% of cases, which have also been found in CD patients, involved in the interleukin signaling pathway. Other pancreatic cancer-related altered genes feature *KIAA1109,* associated with susceptibility to celiac disease and *JAK1* (typical of RCD-2) alterations [25,90,91,134,135]. It is important to keep in mind that celiac disease may co-exist with other autoimmune diseases including diabetes, which may lead to endocrine and exocrine changes, and histopathological alterations to the pancreas [136]. An increased overall risk of pancreatitis has been described in CD patients [137].

### 3.5. Thyroid Neoplasms

Volta et al. found that celiac patients carry a 2.5-fold increased risk of thyroid papillary cancer, stating that the early diagnosis of CD and strict adherence to a gluten-free diet did not have a protective effect on the development of this malignancy [138,139]. Several studies indicating the risk of thyroid papillary cancer development in CD patients may suggest an additional thyroid examination once the diagnosis of CD is established [138,139].

### 3.6. Gynecologic, Breast, and Other Malignancies

Interestingly, patients that have been diagnosed with CD also have a decreased risk of breast cancer (HR, 0.83; 95% CI, 0.74–0.92), endometrial cancer (HR = 0.60; 95% CI = 0.41–0.86), ovarian cancer (HR = 0.89; 95% CI = 0.59–1.34), and lung cancer (HR, 0.88; 95% CI, 0.75–1.03) [12,17,74,140,141].

The peculiar data, though, are an identification of *GATA3* SNP identified in CD by Immunochip data meta-analysis, considering that alterations of this gene are noted in about 17% of breast cancer [26,90,91,142].

The decreased risk of female cancers (breast, endometrial, and ovarian) in CD patients is explained by a probable low estrogen exposure, generally reduced in the lifetime of celiac patients, and the early menopause that celiac patients may experience if not treated properly [140].

The decreased risk of breast cancer specifically in CD could be attributed to a generally lower body mass in CD patients due to decreased nutritional status and malabsorption [12,88]. Other studies indicate a role of concurrent lactose intolerance of CD patients [17,143], thus reducing milk products consumption, which are known to contain the insulin-like growth factor 1 (IGF-1) that may promote tumorigenesis due to the reduction in apoptosis and angiogenesis promotion [144].

The decreased risk of lung cancer can be related to a lower smoking incidence in this population [12].

Despite the known possibility of skin lesions in CD patients, no association with cutaneous melanoma has been detected [145].

**Figure 2:**
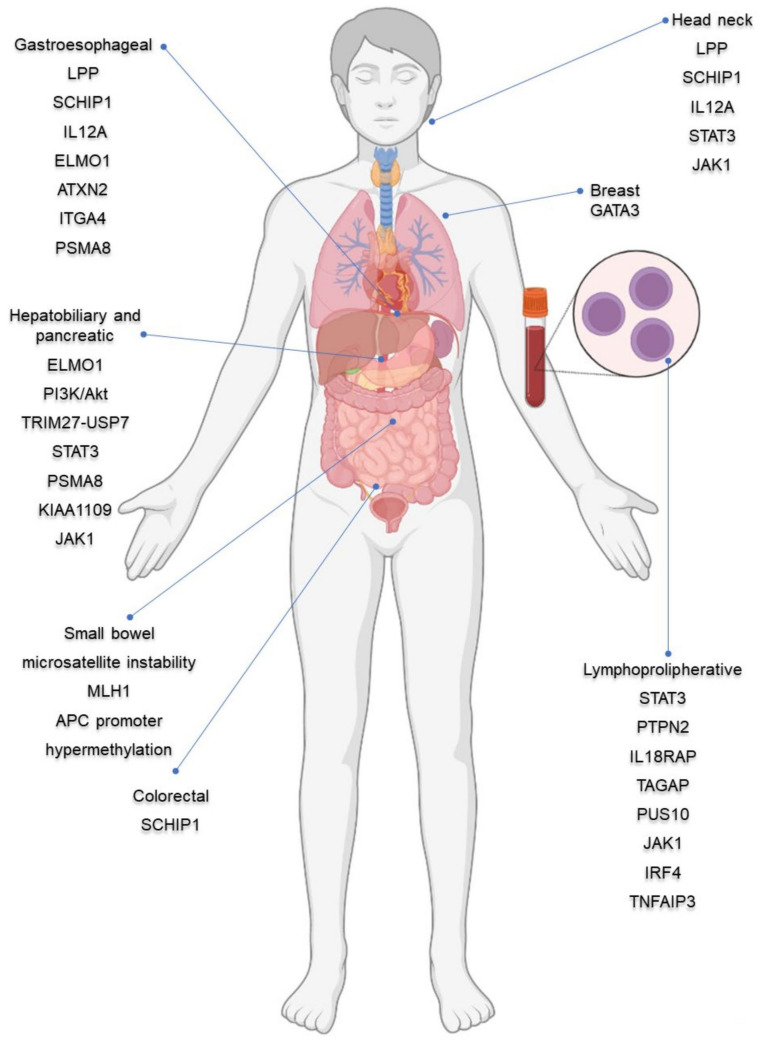
Celiac-disease-related gene alterations commonly shared with cancer pathways, graphically summarized according to Table 1 and paper sections. Created with BioRender.

The complete list of cancer hallmarks commonly shared with celiac disease pathogenesis and malignancy types with increased incidence in celiac patients sharing common gene and molecular alterations is shown in Table 1 and schematically represented in Figure 2.

## 4. Screening, Treatment, and Prevention

Most national and international CD guidelines advise screening in high-risk groups, including first-degree relatives of patients with CD and those with associated high-risk disorders [11]. The diagnostic algorithm is referred to serological testing, followed by a biopsy in the case of a positive result [11]. Recent guidelines also suggest avoiding duodenal biopsies in children with a clear clinical presentation and positive serology [146]. In seronegative patients who refuse to undergo endoscopy, genetic tests for the absence of human leukocyte antigen haplotype HLA-DQ2/8 may be useful to exclude CD [83].

Monitoring the patients with CD-specific antibodies or measurement of gluten immunogenic peptides in urine and feces may be useful in patients with early CD diagnosis to assess recent gluten exposure and predict the absence of histological lesions, thus preventing disease progression [147].

The role of a gluten-free diet, being the only proven effective treatment, is to conserve the mucosa integrity, avoiding CD-related inflammation and atrophy, which may play role in future carcinogenesis [1,8,38], and, in the presence of RCD, evaluate these patients at early stages and prevent them from undergoing a transition from RCD1 to RCD2, given the potential risk of EATL development [1,38]. However, some authors suggest that the capability of intestinal mucosa to restore itself in CD is only partial [42]. The possibility of gut microbiota transplantation has been considered; however, it is still unclear which microorganisms should be selected and safely transferred for the benefit of the patient [45,53]. The lack of diet compliance by CD patients may become a major limitation in correct CD and RCD differential diagnosis, making CD-specific antibodies a cornerstone investigation in this instance [38].

In addition to nutritional support, various therapies have been proposed, such as immunotherapy, but have been found useful only in RCD-1, and hematopoietic stem cell transplantation following high-dose chemotherapy as an alternative treatment [1,38,148]. Phase II “gluten-challenging” trials have shown glutenase ALV003 [149,150], latiglutenase IMGX003, and acetate AT1001 [151,152] to be able to attenuate gluten-induced small intestinal mucosal injury in patients with celiac disease, but there is no widely adopted implementation observed for these drugs, and some trials are ongoing.

Genetical screenings could be powerful predictive instruments for CD patients, for example, the identification of somatic mutations in *JAK1* and *STAT3* could be useful to predict the risk of EATL development in patients with RCD2 [1,38,39].

The overall higher rate of gastrointestinal disorders due to digestion issues give rise to awareness of the control of malabsorption, gastroesophageal reflux, and colorectal adenomas, especially left-sided [17,74,104,115,124]. Pancreatic and liver lesions could possibly be prevented by the careful monitoring of biochemical bloodwork [12,17,129]. The risk of small bowel adenocarcinoma development, even relatively low, should be carefully considered, being higher in CD patients compared to healthy individuals [105,119]. Data on higher tumor-infiltrating lymphocyte (TIL) numbers in small bower adenocarcinoma in CD patients could probably be linked to refractory CD type and could be proposed as a prognostic factor together with MSI instability upon prospective investigations [122].

A small number of studies, uncovering the increased risk of thyroid papillary cancer development in CD patients, prompted clinicians to a careful thyroid examination in all CD patients to develop effective prevention strategies [138,139].

Even though the risk of breast, endometrial, and ovarian cancers is low in CD patients, they should not remain unconsidered if hormone replacement therapy is administered [17,140].

In the context of the overall population, we should probably pay more attention to the elderly population, for both naturally acquired risk factors and possible late CD diagnosis [2,8,12,17,74,75,76,153].

Overall, in addition to following the gluten-free diet, all CD patients are given the widely accepted recommendations of a healthy lifestyle preventing cancer risks, such as high fiber consumption, physical activity, healthy body mass, and smoking cessation [12,88,154].

## 5. Discussion

An increased cancer incidence in patients diagnosed with CD has been found in many studies [8,12,17]. Most of these studies, however, were based on data from patients that were diagnosed before widespread serologic testing, so the cohort possibly represented a more severe disease phenotype [12,155,156]. One should not overlook the possible delayed seropositivity of CD, which can be caused by co-occurring and/or autoimmune diseases, as described in a case report by Kostopoulou et al. in patients with type 1 diabetes mellitus [157]. It is important to remember that CD may be associated with other autoimmune comorbidities, potentially increasing the risk for certain malignancies [88].

Some authors claim that, even though CD represents an increased risk of gastrointestinal cancers due to possible disease latency and chronic inflammation, the risk is high in the first year after diagnosis with little to no risk thereafter [12,155]. These data may be explained by the fact that many studies were conducted in subjects with CD prior to the serological testing, so a long time passed before the adoption of a gluten-free diet, which has been shown to mitigate autoimmune inflammatory response and, therefore, reduce overall risk [12,80].

Another explanation may be supported by the data of esopageal and small bowel cancer incidence in CD patients, where the risk was observed to be higher in the peridiagnostic period [105]; however, it is not clear if the risk was correlated to pathological mucosal changes in the presence of CD or the diagnosis of malignancy was delayed by its silent nature, especially typical of small bowel carcinoma.

Recent studies also emphasized that gluten intake is not associated with cancer risk in adults without CD, and for those people, dietary gluten restriction is unlikely to play a preventive role in cancer development [158,159]. On the contrary, inconsistency in the gluten-free diet in celiac patients may arise numerous inflammatory conditions, which unfavorably contribute to possible carcinogenesis. Thus, it is possible that a gluten-free diet may minimize the risk of cancer where inflammation is a major trigger [23,129,158,160].

The abovementioned higher risk of malignancies in patients diagnosed with CD after the age of 60 years [12] could be connected with the fact that, despite most diagnoses of CD are at present made in children and young adults, there are still 20–30% of celiac patients who were first diagnosed at age over 60 years in several countries such as Canada, the United States, and Northern Europe [87,153]. These data, indeed, leave an open question, of whether CD develops at an advanced age or remains undiagnosed during one’s lifetime [87], although it is known that patients diagnosed with CD in older age tend to be seronegative [83]. Other reasons could be the overall worldwide population aging and the increased risk of cancer promoted by DNA replication errors accumulating during one’s life, overall higher malignancy rate in the elderly, as well as the diminishing capacity of mucosa healing in the context of possibly low adherence to a gluten-free diet [87,88,161].

## 6. Conclusions and Future Perspectives

The effect of a gluten-free diet in CD patients is unequivocally helpful for reducing the main symptoms of the disease and improving the quality of life of the patient [10]. The effect of this diet on reducing or preventing the development of malignancies in those patients is still debatable [17].

Patients diagnosed with CD should be aware of slow mucosal healing in the case of non-strict diet adherence, and the process may be utterly slowed down in the presence of concomitant diseases or aging and are recommended to undergo a strict follow-up during different periods of the disease [154,162]. While the majority of patients respond to a GFD, up to 20% of patients with CD have persistent or recurrent symptoms [146].

Nevertheless, the awareness of the CD diagnosis and treatment has substantially grown in recent decades, and there are still many factors to discover in the risk of development of CD-associated neoplasms [8]. Expanding our knowledge on the complex nature of CD suggests the improvement of early disease detection and sensitive biomarkers establishment. Genomics, epigenomics, and transcriptomics development have significantly amped the overview of the CD immune landscape [42]. The possible future validation of non-invasive tests, such as miRNA detection in the blood and saliva, may significantly ease the diagnostic process [20,69].

RNA sequencing has recently allowed researchers to create a map of microbial biomarkers along the gastrointestinal tract for celiac disease patients, which also describes the effect of gluten-free diet [163]. A new study by Khalkhal found the mRNA expression of six genes, suggesting some of them are useful and sensible markers in differentiating patients with celiac disease from healthy controls [164]. Circulating miRNAs, such as miR-192, could represent important biomarkers in clinical practice, and therefore are an exciting target, but require further studies [72].

DNA methylation assays are new promising lines of research. Peng et al. recently performed a plasma-based multiplex DNA methylation assay of a plethora of upper gastrointestinal cancer samples, resulting in three methylated cancer-specific signatures [108]. The methylated *ZNF582* and *TFPI2* and *ELMO1* have been proposed as an alternative panel for the early detection of non-invasive upper gastrointestinal cancer [108]. Given that the latter has been found deregulated in inflammatory bowel disease and proposed as a potential early biomarker, this finding could evolve into a potent diagnostic instrument for CD and gastrointestinal cancer risk assessment [109].

Numerous kinds of research suggest various methylation and gene expression profiles as novel non-invasive CD diagnosis tools [65,66].

The screening for somatic mutations in *JAK1* and *STAT3* found in CD patents with RCD2 [1,38,39] could become a potential therapeutic target for RCD2 treatment, blocking progression toward EATL [39]. The emerging results of the single-cell mass cytometry of RCD2 patients highlighted intertumoral and intratumoral cell heterogeneity within the duodenal and peripheral aberrant cell population, which may offer a clue to the therapy responsiveness upon further investigations [165].

Microsatellite instability (MSI), another important tumorigenesis factor, may as well become a potential target in CD patients. MSI is a hallmark and a surrogate test of mismatch-repair (MMR) deficiency, which has been observed in CD patients with small-bowel carcinoma [122]. A recent retrospective study of gastrointestinal tract cancers has revealed that 34% of small intestinal cancers are MMR-deficient [166]. Immunohistochemical (IHC) testing for four mismatch-repair (MMR) protein (MLH1, MSH2, MSH6, and PMS2) expression to date is the most cost-effective method to evaluate MMR status [167], so it would be interesting to assess the MMR status in CD patients without any identified tumors, given that CD shares many common cancer hallmarks. This could lead to a possible new immunotherapy application [168]. To our knowledge, there has been no study assessing MMR status in CD [166].

The presence of tumor-infiltrating lymphocytes (TILs) has a positive predictive value in many cancer types’ treatment and prognosis [43,169,170]. These include T lymphocytes, containing programed cell death 1 (PD-1) protein, which interacts with the programed cell death ligand 1 (PD-L1) located on the surface of neoplastic cells, leading to the decrease in anti-tumor immune response [171]. Considering the findings of Vanoli et al. [122], the higher TIL number in small bower cancers in CD patients could propel a new research line of the PD-1/PD-L1 axis in CD patients.

The future discovery of the diseases’ epigenetics may complete the clarification of the nature of CD, thus improving its prognosis, averting complications, and understanding the association with the risk of cancer development [8,20,26].

Increasing the number of retro- and prospective studies around the world could probably make a significant contribution to the understanding of the diseases’ biology and pathogenesis.

## Figures and Tables

**Table 1:** Cancer hallmarks commonly shared with celiac disease pathogenesis, and genetic/molecular alterations commonly observed in celiac disease and different malignancy types.

Group of Disorders	Role	Hallmarks/Genetic and Molecular Alterations	Ref.
Cancer hallmarks	Epithelial transdifferentiation	Inflammation	[18]
	Chromosome alterations	Genome instability	[18]
	Epigenomic modifications, cell reprogramming	Genome mutations	[18]
	Epithelial dedifferentiation	Phenotypic plasticity	[18]
	Invasiveness	Epigenetic reprogramming	[18]
	Tumor growth, immune evasion, therapy resistence	Polymorphic microbiomes	[18]
Lymphoproliferative	Interleukin signalling, DNA repair	*PSMA8*	[25]
	Malignant transformation	*JAK1*	[38,39]
	Malignant transformation	*STAT3*	[38]
	Malignant transformation	*PTPN2*	[90,91]
	Malignant transformation	*IL18RAP*	[90,91]
	Malignant transformation, tumor reccurence	*TAGAP*	[90,91]
	Malignant transformation	*PUS10*	[90,91]
	Immune response and cell proliferation regulations	*IRF4*	[96]
	Malignant transformation	*TNFAIP3*	[39]
Head and neck	Tumor cell migration, invasion, and metastasis	*LPP*	[90,91]
	Malignant cell proliferation	*SCHIP1*	[90,91]
	Antitumor immunity	*IL12A*	[99,100]
	Malignant transformation	*STAT3*	[38,101]
	Malignant transformation	*JAK1*	[38,102]
Gastroesophageal cancer	Tumor cell migration, invasion, and metastasis	*LPP*	[17,90,91]
	Malignant cell proliferation	*SCHIP1*	[17,90,91]
	Antitumor immunity	*IL12A*	[17,90,91]
	Cancer invasion, metastasis	ELMO1	[90,91,107]
	Malignant transformation	*ATXN2*	[28]
	Malignant cell migration	*ITGA4*	[113,114]
	Interleukin signalling, DNA repair	*PSMA8*	[25]
Small bowel cancer	Microsatellite instability, impaired DNA repair	MLH1 methylation	[122]
	Impaired DNA repair	Microsatellite instability	[120]
	Tumor supressor, cell migration, apoptosis	*APC*	[60,90]
Colorectal cancer	Malignant cell proliferation	*SCHIP*	[128]
Hepatobiliary and pancreatic cancer	Malignant cell proliferation and survival	PI3K/Akt	[132]
	Malignant cell promotion	TRIM27-USP7	[133]
	Malignant transformation	*STAT3*	[38,133]
	Interleukin signalling, DNA repair	*PSMA8*	[25]
	Cancer invasion, metastasis	ELMO1	[107,132]
	Cancer invasion, metastasis	*KIAA1109*	[25,90,91,134,135]
	Malignant transformation	*JAK1*	[38]
Breast cancer	Cell differentiation	*GATA3*	[26]

## Data Availability

No new data were created or analyzed in this study. Data sharing is not applicable to this article.
